# The eSNV-detect: a computational system to identify expressed single nucleotide variants from transcriptome sequencing data

**DOI:** 10.1093/nar/gku1005

**Published:** 2014-10-28

**Authors:** Xiaojia Tang, Saurabh Baheti, Khader Shameer, Kevin J. Thompson, Quin Wills, Nifang Niu, Ilona N. Holcomb, Stephane C. Boutet, Ramesh Ramakrishnan, Jennifer M. Kachergus, Jean-Pierre A. Kocher, Richard M. Weinshilboum, Liewei Wang, E. Aubrey Thompson, Krishna R. Kalari

**Affiliations:** 1 Division of Biomedical Statistics and Informatics, Mayo Clinic, Rochester, MN 55905, USA; 2 Division of Clinical Pharmacology, Department of Molecular Pharmacology and Experimental Therapeutics, Mayo Clinic, 200 First Street SW, Rochester, MN 55905, USA; 3 Fluidigm Corporation, South San Francisco, CA 94080, USA; 4 Department of Cancer Biology, Mayo Clinic, 4500 San Pablo Road, Jacksonville, FL 32224, USA

## Abstract

Rapid development of next generation sequencing technology has enabled the identification of genomic alterations from short sequencing reads. There are a number of software pipelines available for calling single nucleotide variants from genomic DNA but, no comprehensive pipelines to identify, annotate and prioritize expressed SNVs (eSNVs) from non-directional paired-end RNA-Seq data. We have developed the eSNV-Detect, a novel computational system, which utilizes data from multiple aligners to call, even at low read depths, and rank variants from RNA-Seq. Multi-platform comparisons with the eSNV-Detect variant candidates were performed. The method was first applied to RNA-Seq from a lymphoblastoid cell-line, achieving 99.7% precision and 91.0% sensitivity in the expressed SNPs for the matching HumanOmni2.5 BeadChip data. Comparison of RNA-Seq eSNV candidates from 25 ER+ breast tumors from The Cancer Genome Atlas (TCGA) project with whole exome coding data showed 90.6–96.8% precision and 91.6–95.7% sensitivity. Contrasting single-cell mRNA-Seq variants with matching traditional multicellular RNA-Seq data for the MD-MB231 breast cancer cell-line delineated variant heterogeneity among the single-cells. Further, Sanger sequencing validation was performed for an ER+ breast tumor with paired normal adjacent tissue validating 29 out of 31 candidate eSNVs. The source code and user manuals of the eSNV-Detect pipeline for Sun Grid Engine and virtual machine are available at http://bioinformaticstools.mayo.edu/research/esnv-detect/.

## INTRODUCTION

The advent of next generation sequencing technologies has revolutionized both basic science and medicine; comprehensive understanding of genomic and transcriptomic variants provides clues to novel biological mechanisms and molecular basis of complex diseases (1,2). In particular, discovery of single nucleotide variants (SNVs) from genomics and transcriptomics plays a significant role for treatment of disease (3). Germline and somatic SNVs from genomics and transcriptomics sequencing studies in cancers have allowed us to define mutational landscape of tumors (4–7). Most of the studies derive SNVs from targeted approaches, but transcriptomics allows us to obtain SNVs in an unbiased manner. As a valuable and cost-effective alternative, transcriptome sequencing or RNA-Sequencing (RNA-Seq) has attracted much attention, because it helps obtain a variety of genomic features from a single high throughput experiment. For example, genomic features such as gene expression, transcript expression, novel isoforms, fusion transcripts, expressed single nucleotide variants (eSNVs), circular RNAs, non-coding RNAs (long non-coding RNAs and small RNAs) can be obtained from RNA-Seq data (8). Well-developed analytical methods are available for obtaining gene expression counts, transcript counts and fusion transcripts from RNA-Seq data (9–11). However, no robust bioinformatics pipelines exist for identification of eSNVs in the transcriptome data.

Several cancer studies have shown that novel and somatic SNVs can be obtained by sequencing normal and tumor tissue from the same individual (12,13). Specifically, the somatic point mutations identified could be essential driver mutations for tumorigenesis (14,15). To date, large-scale studies have used exome sequencing to call germline and somatic SNVs from cancer (4–7). However recent studies revealed that only 20% of the SNVs overlap from multiple variant-calling pipelines indicating the challenges of calling variants from exome sequencing (16). As another independent source of information, calling SNVs from RNA-Seq can be beneficial, because it allows us to investigate the mutations from a different sequencing based approach. Considering the low tumor purity in cancer DNA, RNA-Seq may be used to detect even low-frequency mutations that are expressed, and which may be difficult to detect using exome sequencing.

Computational methods to discover SNVs from RNA sequencing compared to exome sequencing are underrepresented in bioinformatics. Only two groups developed methods and have made their tools publically available to call eSNVs from RNA-Seq (17,18). These methods either lack potent filtering steps to remove false positives or require merging of multiple samples to obtain extreme high coverage for accurate variant calling. Further, both methods require a single aligner, which inevitably introduces systematic bias. Although these methods successfully brought RNA-Seq into the practical application of genomic variant calling they have had specific applications to certain diseases (19).

A critical step for variant calling from RNA-Seq data is the read alignment to the whole genome/transcriptome. There are several aligners that are currently available for RNA-Seq alignment (20–24). Each aligner has its own strengths and weaknesses and it is difficult to choose a single aligner that is both efficient and proficient for RNA-Seq data. The choice becomes trivial when examining regions with high read depth and/or low genomic complexity, as most variant callers will agree in the calling results. However, Engstrom and their group recently compared and summarized 26 RNA-Seq alignment protocols and have shown that the disagreements of aligners are often due to a fraction of reads that are highly mutated or due to reads that map to splice junctions (25). It is the challenges of the low coverage and/or region of high genomic complexity that motivate each aligner to devise different alignment strategies: mismatch and gap placement, dealing with transcript reconstruction, genomic repeats and pseudogenes, etc. The eSNV-Detect system was designed to leverage the different evidence from multiple aligners to increase the confidence level of variant calls.

In addition, published bioinformatics eSNV algorithms have focused only on variant identification, while the follow-up analyses including determination of variants effects at a protein domain still require a substantial amount of effort. In this context, we have developed an eSNV calling method that can be used to call variants confidently in a clinical setting, with complete annotation and easy prioritization of variants for functional follow-up analysis. At Mayo Clinic, we have applied our variant calling method for a variety of cancer and other disease related datasets (26,27). However the method can be applied to other species by providing the pipeline with the appropriate reference genome of interest. The pipeline can now be executed on a stand-alone UNIX machine or a parallel computing environment with sun grid engine (Oracle Corporation, Redwood City, CA). A virtual machine version of the eSNV-Detect pipeline is also provided for users without access to a UNIX workstation. The pipeline is publicly available for download at http://bioinformaticstools.mayo.edu/research/esnv-detect/.

In this manuscript, we have provided details of parameters used for developing the eSNV-Detect method and have presented a variety of analyses to show the accuracy of variant calling from non-directional paired-end RNA-Seq data. To determine sensitivity and specificity of the eSNV-Detect method, we have investigated the variants from a set of 25 The Cancer Genome Atlas (TCGA) ER+ breast tumors for which we have RNA and exome sequencing datasets along with a 1000 genome individual for whom we have RNA-Seq and single-nucleotide polymorphisms (SNP) chip dataset. We have also obtained eSNVs for an MD-MB231 cell line sample from the COSMIC (28) database and examined those variants from single-cell RNA-Seq data and whole transcriptomic RNA-Seq dataset. The robust accuracy metrics obtained in calling eSNVs will allow us to perform future functional validation of the variants or perform allelic specific expression or quantitative trait loci studies precisely. Most of the published methods stop at *in-silico* nomination of the variants and do not perform any extensive validation of the variants using Sanger sequencing or other functional experiments. In our case, we have shown proof of principle of the eSNV-Detect method by identifying the list of novel eSNVs called with RNA-Seq data and validated them using Sanger sequencing with high accuracy (79/83 variants) from a tumor and adjacent normal breast sample (27). We have also provided here Sanger sequencing validation results for an estrogen receptor positive (ER+) breast tumor and adjacent normal tissue from the same individual.

## MATERIALS AND METHODS

### The eSNV-detect pipeline

#### Variant calling and filtering

**Figure 1.**
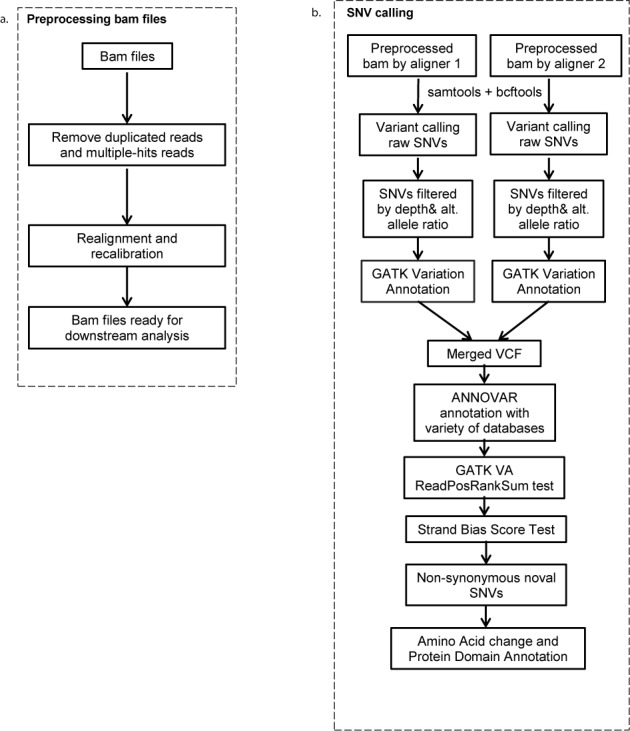
Flow chart of the eSNV-Detect pipeline. (**a**) Bam files are pre-processed by Picard and GATK to remove reads that are duplicated or have multiple hits. (**b**) SNVs called from two aligners are merged, annotated and filtered by genetic features like total read depth, alternative allele depth and frequency, as well as annotations.

Figure 1 shows the flowchart of the eSNV-Detect workflow, which will work with non-directional paired-end RNA-Seq data. Bam files generated by two aligners are refined through a pre-processing step to remove reads that are polymerase chain reaction (PCR) duplicates and those mapped to multiple regions of the genome. Remaining unique mapped reads from the pipeline are realigned and recalibrated using Genome Analysis Tool Kit (GATK) as shown in Figure 1. Samtools mpileup and bcftools with filtering criteria of base quality >13 and mapping quality >20 are used to call variants from the realigned and recalibrated bam files. To obtain sensitive variant calling, other parameters of samtools mpileup and bcftools are turned off. To minimize false negative and false positive variant calls, we apply a set of thresholds as described in the following section. Nucleotide positions with <4X coverage or four alternative allele supporting reads are eliminated from variant analysis. Ratio of reads at a nucleotide position is calculated by Ratio*_i_* = aa_*i*_/*t_i_* (where i is the nucleotide base pair, aa is the alternate read depth at the location i and t is the total number of reads at location i). For variants with total coverage <100, we require the Ratio_*i*_ to be >0.1; while for variants with higher coverage (≥100), the Ratio*_i_* threshold should be 0.05. We reduce the threshold for high coverage positions to enhance the sensitivity to detect lower-frequency mutation events, keeping the tumor clonality issue in mind. In addition to frequency filters, we also apply strand bias filter, which is defined as the ratio of forward strand alternative allele counts (aa*_i_*+) and the reverse strand alternative allele (aa_*i*_−) counts, whichever greater will be the denominator. For variants with total coverage (*t_i_*) <100, the strand bias ratio (SBS_*i*_) of >0.1 is required and for variants with >100X coverage a SBS*_i_* of >0.05 is preferable. In order to exclude false positives with most of the aa*_i_* supporting reads located at the 5′ or 3′ end of the reads, we obtain the ReadRankPosSum (RRPS) score using the GATK. A recommended RRPS score threshold of (−8.0, 8.0) from GATK is used in our method. The set of read characteristics used as filters are summarized in Table 1. After filtering, the variant calling files from two aligners are merged and annotated. Priority is assigned to each variant according to the two-aligner strategy as discussed below.

**Table 1.** The eSNV criteria used in the eSNV-Detect pipeline

Criteria	Threshold
Alternative allele supporting read depth	d_alt>3
Alternative allele frequency	if (total read depth >100) alt/ref >0.05 else alt/ref>0.1
Strand bias ratio	if (total read depth >100) alt/ref >0.05 else alt/ref>0.1
ReadRankPosSum	-8<RPKS<8

#### Two-aligner strategy

In the eSNV-Detect, we have chosen the multi-aligner concept to call the variant confidently. Our previous work has shown that using multiple aligners to call variants brings complementary strengths and reduces false discovery rates significantly (27). We define the confidence and assign priority (high, medium and low) to a candidate eSNV call based on the concordance and the alignment source of the variant. High confident variants (labeled as CONF = 2 in the output) are those identified by two aligners and retained even after filtering steps, which indicated less genomic ambiguity for such variants between different aligners. Lower confident variants (labeled as CONF = 1 or 0) are defined as those variants with evidence from only one aligner, reflecting the disagreement between different aligners and thus more likely to be a false positive caused by mapping error. The user is allowed to assign a preferred aligner, and the variants with evidence only from the preferred aligner are assigned with medium confidence (CONF = 1). Those from the less preferred aligner are assigned with low confidence (CONF = 0).

#### Annotation

The ANNOVAR software is used to annotate the variants using dbSNP, 1000 genome, 5400 exome, refGene, avsift and ljb_phylop from UCSC databases (29,30). Further downstream annotations of non-synonymous variants were obtained by mapping the Refseq identifiers of the amino acid alterations to their corresponding UniProt (31) identifiers and annotating them with the human proteome (59052 human proteins) using HMMER (32) and Pfam 26.0 (November 2011, 13 672 families) (33). The in-depth annotations of variants enable ease and convenience to explore the expressed SNVs that may structurally or functionally impact protein domains. This further enables prioritization of eSNVs for downstream functional and clinical investigations.

### 1000 genome sample

We obtained transcriptomic sequencing and SNP chip data for a lymphoblastoid cell line (NA07347) from the 1000 genome project (34,35). The RNA-Seq data was generated by Genome Analyzer II (Illumina, San Diego, CA) with a total read depth of 67 M paired-end reads (75 bp). The SNP-chip data was generated by HumanOmni2.5 BeadChip (Illumina).

### Sanger sequencing validation of variants

For Sanger sequence validation, we obtained one ER+ breast tumor and matched normal tissues for which we have variant calls from RNA-Sequencing of the tumor. Variants predicted by the eSNV-Detect in the ER+ tumors were validated by Sanger sequencing at the Mayo Clinic. Primer3 version 4.0 software was used to deign primers for the variants. The extracted DNA was amplified by PCR and purified, 1.6 pM of one of the primers (forward or reverse) and 80 ng of the purified PCR product was used for sequencing. Electropherograms obtained were analyzed using SeqScape v2.5 (ABI, Applied Biosystems, Foster City, CA, USA).

### *In-silico* validation of TCGA exome and RNA-Seq data

To validate the eSNV-Detect method, we randomly selected 25 TCGA ER+ breast tumors and downloaded the sequencing bam files via the CGHub data portal (https://cghub.ucsc.edu/). The list of 25 TCGA samples including their exome and RNA sequencing depth and mapped reads are listed in Supplementary Table S1. The original TCGA RNA-Seq data was aligned using MapSplice (23) and TopHat (version 1.3), using fastq files converted from the MapSplice bam files using in-house scripts. The two bam files were then reprocessed and recalibrated by the eSNV-Detect workflow to call variants. The variants from 25 RNA-Seq ER+ tumors were further annotated and compared with the corresponding 25 TCGA ER+ breast tumor exome sequencing data to validate the eSNVs called for each sample.

### Single cell sequencing of a breast cancer cell line

MDA-MB-231 breast cancer cell line (ATCC HTB-26) was cultured in Leibovitz's L-15 medium with 10% fetal bovine serum for 5 days. Duplicate cultures were processed for single-cell analysis. Single-cell were captured on a large-sized (17–25 um cell diameter) microfluidic mRNA-seq chip known as the C1™ Single-Cell Auto Prep IFC, using the C1™ Single-Cell Auto Prep System (Fluidigm Corporation, South San Francisco, CA). Cells were loaded onto the chips at a concentration of 300 cells/μl, stained for viability with LIVE/DEAD cell viability assay kit (Life Technologies, Carlsbad, CA) and imaged by phase contrast and fluorescence microscopy to assess the number and the viability of cells per capture site. Only 16 single, live cells were included in the analysis. cDNAs were prepared on chip using the SMARTer Ultra Low RNA kit for Illumina (Clontech Laboratories, Mountain View, CA). Single-cell cDNA size distribution and concentration was measured with Quant-iT Pico green dsDNA assay kit (Life Technologies). Illumina libraries were constructed in 96-well plates using the Illumina Nextera XT DNA Sample Preparation kit using the protocol supplied by Fluidigm. Libraries were quantified by Agilent BioAnalyzer, using high Sensitivity DNA analysis kit. Single-cell Nextera XT (Illumina) libraries of one experiment were pooled and sequenced at 100 bp paired-end on Illumina Miseq to a depth of about 150 000 reads. For validation of the variants called from MiSeq data we have processed the RNA-Seq data that is publically available, from Gene Expression Omnibus (GSE27003), from whole transcriptome sequencing of the same MDA-MB-231 cell line (36). The traditional transcriptomic data of MDA-MB-231 was sequenced by Illumina GA-II platform with more than 59 million reads. Both single-cell mRNA-Seq data and traditional GA-II mRNA-Seq data were processed through the eSNV-Detect pipeline using TopHat2 and Burrows-Wheeler Aligner (BWA) aligners. To account for the low sequencing depth of MiSeq platform, the alternative allele read depths threshold for the single-cell sequencing data was reduced to two sequence reads in the eSNV-Detect pipeline.

### Performance evaluation

The performance of the eSNV-Detect was assessed in terms of variant precision and recall. We used whole genome SNP chip data (HumanOmni2.5 BeadChip) from 1000 genome project and whole exome sequencing (WES) data from TCGA ER+ breast tumors to validate eSNVs. True positive (TP) was defined as those identified by the eSNV-Detect and validated by the Omni 2.5 genotyping or WES data. False positive (FP) was defined as variants that were identified by our method, but not confirmed by the Omni/WES data. False negative (FN) was defined as the variants found in Omni/WES data, but not identified by the eSNV-Detect method. In the case of the 1000 genome sample, since Omni chip covers only a portion of the whole genome, we validated eSNVs based on the 2.4 million locations. Since all reference bases were the true negatives (TN), instead of specificity, we chose to calculate precision to evaluate the performance of our method by using the below formula: precision = TP/(TP + FP). Recall, or sensitivity, was calculated as: recall = TP/(TP + FN).

## RESULTS

A novel computational pipeline (the eSNV-Detect) was developed to identify known and novel expressed SNVs from RNA-Seq experiment. To call variants the software requires post alignment files from any two aligners. The two aligner concept has been shown to be effective in reducing the false positives (27). Below are few examples of how we have shown the utility of the software in a lymphoblastoid cell line, Sanger validation of an ER+ tumor sequenced at Mayo, TCGA ER+ breast tumors and single-cell RNA-Seq data from a breast cancer cell line (Supplementary Methods). The mapping strategies used in the below examples are BWA + TopHat2 for most of the analyses (lymphoblastoid cell line, Mayo ER+ tumor samples and single-cell data from breast cancer cell line). We applied TopHat + MapSplice combination only for 25 TCGA ER+ breast tumors. We chose this combination, because all the TCGA RNA-Seq data from TCGA data repository has MapSplice alignments readily available.

### High precision of the eSNV-detect method when applied to a lymphoblastoid cell line

We applied the eSNV-Detect method for the RNA-Seq data of a lymphoblastoid cell line (NA07347) from the 1000 genome project. Alignment of the RNA-Seq data was performed by TopHat2 and BWA against the human genome (release NCBI GRCh37.1b) respectively and the bam files were processed through the eSNV-Detect pipeline. In this analysis, we chose the splice aligner TopHat2 as the preferred aligner. The variant calls from the workflow were validated with the HumanOmni2.5 SNP chip that consisted of genotyping information for 2 448 222 genomic locations over the whole genome.

**Figure 2.**
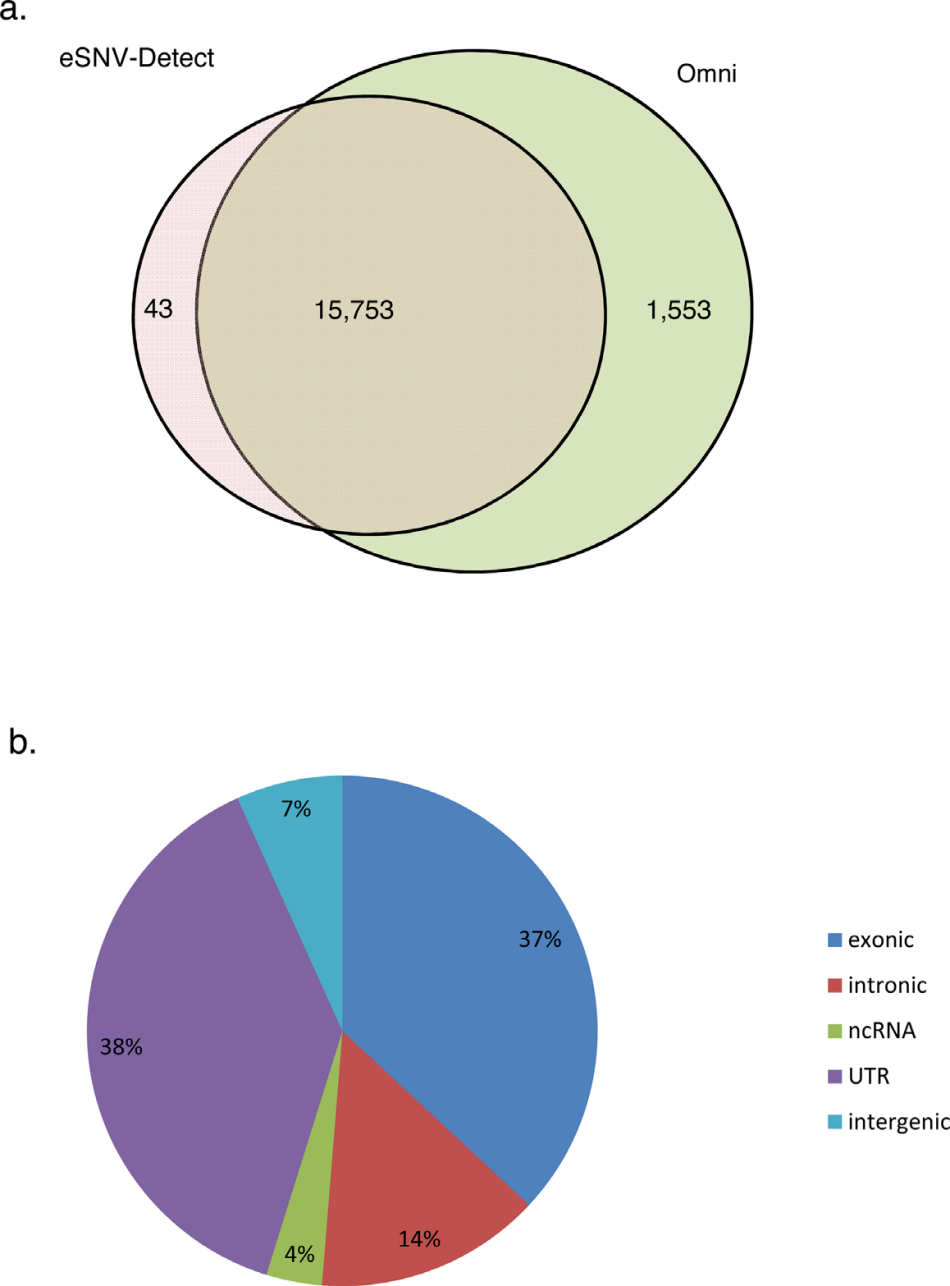
Validation of the eSNVs in NA07347 mRNA-Seq data against the Omni 2.5 Chip data. (**a**) 15 753 out of 15 796 eSNVs were validated by the Omni data. There were 1554 Omni SNPs that were expressed but not called by the eSNV-Detect; (**b**) The validated 16 441 validated eSNVs distributed across the whole genome, mainly in exonic (36.9%), UTR (38.4%), intronic region (14.3%).

Our method identified 39 255 high confident (validated by both aligners, CONF = 2) eSNVs in the NA07347 RNA-Seq data, of which genotyping data was available for 15 796 nucleotide positions on the HumanOmni2.5 chip. The remaining eSNVs could not be validated due to absence of genotype information. Hence, our validation was based on these 15 796 loci. The HumanOmni2.5 chip data confirmed 15 753 out of the 15 796 RNA-Seq eSNV candidates to be true positives and the eSNV-Detect achieved a high precision rate of 99.7% (Figure 2a). The genomic composition of the 15 753 validated eSNVs is shown in Figure 2b. The variant calls were mainly present in exonic and untranslated region (UTR) regions, but part of the high precision calls were also distributed in intronic and intergenic regions.

Of the 2 448 222 SNP loci on the Omni chip of NA07347, only 17 307 SNPs were expressed in the transcriptome (i.e. >3 alternative allele supporting reads in the RNA-Seq data. A detailed transcriptomic expression distribution of all SNPs on the Omni chip can be found in Supplementary Table S2). Among the expressed variants, the eSNV-Detect called 15 753 out of 17 307 as high confident eSNVs and achieved a high sensitivity/recall rate of 91.0%. The 1553 variants not found in the high confident eSNV list were either called by only one aligner (683 found with TopHat evidence only and 19 found in BWA evidence only), or eliminated by the stringent filter criteria (i.e. 851 by low alternative allele frequency or extreme ReadPosRankSum score or strand bias ratio).

We thus also investigated the medium/low confident variants called by single aligner. In the NA07347 RNA-Seq data, there were 4363 medium confidence variants (CONF = 1) with evidence from TopHat2 alone. Among them 706 have genotype information on the Omni and 683 of 703 (97.1%) were verified to be true positive. There were 5106 low confidence variants (CONF = 0) with evidence from BWA only. Among them 358 had genotype information on the Omni Chip and 343 of 358 (95.8%) were validated. As the preferred aligner, eSNVs with only TopHat2 evidence showed a slightly higher precision than those with BWA evidence, while variants set with support from both aligners had the highest precision. Our analysis concludes that the two-aligner strategy improved the precision of the eSNV calling.

### The impact of the selected mapping strategies

Using the same set of data for the lymphoblastoid cell line (NA07347) that consists of both RNA-Sequencing and SNP chip data, we investigated the impact of different mapping strategies. Engstrom and his colleagues (25) have shown that MapSplice, STAR-2pass and TopHat2 are top performance aligners for RNA-Seq. Hence we have chosen these three aligners along with BWA for the following analysis. After alignment with the four aligners, the bam files were processed through the eSNV-detect pre-processing and variant calling steps, respectively. We compared all pair-wise combinations of two-aligners with the Omni SNP chip data. Since the read-depth at a nucleotide position may differ during alignment process, we have chosen SNVs for comparison that have read depth ≥4 in at least two aligners and have Omni-SNP chip data (17389 SNVs).

Among the pair-wise comparisons (Supplementary Table S3), the combination of MapSplice + TopHat2 detected the truest positive variants, thus have the highest recall rate. It should be noted that MapSplice and TopHat2 both use Bowtie (both used bowtie 1 in the comparison) for segment mapping, which could be part of the reason of the high recall rate. It is noted that different combinations of aligners affect the precision very little. We have also tried combinations of three and four aligners to call variants using the eSNV-Detect. Intuitively, the evidence from more aligners may improve the performance precision. However, it turned out that the improvement of precision was only marginal with the price of a substantial loss in recall rate (Supplementary Table S4). Moreover, increasing the number of aligners will require extra computational resources. Hence, we recommend using two-aligner mapping strategy with the eSNV-Detect.

### Sanger sequencing validation of variants identified by the eSNV-Detect in breast tumor and adjacent normal

We have used an earlier version of the eSNV-Detect method to call variants from RNA-Seq data in lung adenocarcinomas (26) and breast cancer samples (27). In a recent study, we have validated the variants predicted by the eSNV-Detect method with high accuracy in ERBB2 overexpressed (HER2+) breast tumors and adjacent normal tissues using Sanger sequencing. In a survey of 32 breast tumors from RNA-Seq data, a HER2+ breast tumor with the highest number of novel eSNVs (83 candidate variants) predicted by the eSNV-Detect was selected for Sanger sequencing validation. Tumor and tumor-adjacent normal tissues were sequenced along with a control sample for validation. We have confirmed 79/83 eSNVs in the HER2+ study using Sanger sequencing (27).

**Figure 3.**
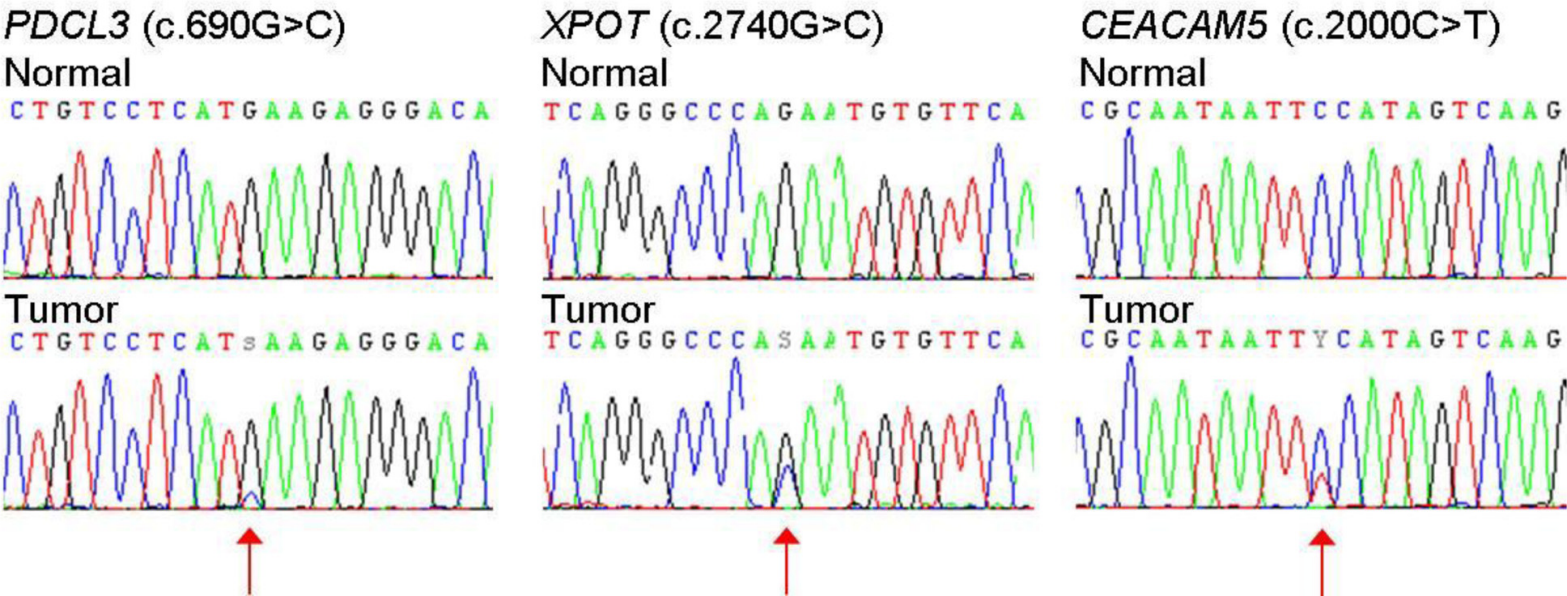
Sanger sequencing validated the eSNVs called. Not only eSNVs with higher allele frequency were validated, an eSNV in PDCL3 gene called with low minor allele frequency was also validated by Sanger sequencing.

Similarly, in the present study, we also selected an ER+ breast tumor sample that was processed through the eSNV-Detect method for validation, and 29 out of 31 eSNVs were validated. An example of Sanger sequence chromatogram plots of eSNVs from ER+ tumor is shown in Figure 3. As indicated in Figure 3A the variant in *PDCL3* gene called with low minor allele frequency and read depth was also validated by Sanger sequencing.

### Validation of eSNVs using TCGA breast cancer exome and RNA-Seq data

MapSplice and TopHat aligned BAM files were obtained to call variants for 25 TCGA ER+ breast tumors. The BAM files from TCGA ER+ tumors were processed through the eSNV-Detect workflow, as described above, to obtain high confident eSNVs (CONF = 2). The number of high confident eSNVs detected ranged from 33 304 to 96 152 in the 25 ER+ tumors. More than 75% of the eSNVs annotated were located in exons, 3′ UTR and 5′ UTR regions and intronic regions, ∼10% of variants were present in non-coding RNAs, and the remaining variants were observed in other regions for the 25 TCGA ER+ breast tumors. High confident eSNVs observed in exonic regions from RNA-Seq data ranged from 7261 to 12 528 as shown in Table 2. It should be noted that the variants were called only from tumors. Thus, these eSNVs consist of both germline and somatic variants. About 40% of the variants were non-synonymous (eSNVs that would result in amino acid changes) and range from 3028 to 6002. Of the non-synonymous eSNVs called, about one-third of them affected protein domains. In total we identified 23 726 unique high-confidence non-synonymous eSNVs that were expressed in the 25 TCGA ER+ tumors investigated. The total read depth of variants called in the 25 ER+ samples had a large dynamic range from 4 to 8009 reads. More than 97% of the 23 736 eSNVs were known single nucleotide polymorphisms that can be found in either 1000 Genomes project or dbSNP databases. High confident non-synonymous eSNVs affecting a domain for these 25 TCGA ER+ tumors are listed in Supplementary Table S5.

**Table 2.** The precision and recall in the 25 TCGA ER+ samples when validated with the protected mutation list from WES data

TCGA sample ID	Validated eSNVs	Total eSNVs	WES No Coverage	Expressed WES SNV	Precision	Recall	F
**TCGA-A2-A04Y**	9464	10 183	231	10 292	0.951	0.920	0.935
**TCGA-A2-A0CT**	8353	9436	534	8838	0.938	0.945	0.942
**TCGA-A2-A0EM**	9608	10 319	251	10 405	0.954	0.923	0.939
**TCGA-A2-A0ER**	9871	10 650	181	10 817	0.943	0.913	0.928
**TCGA-A7-A0CG**	9316	10 733	1058	9868	0.963	0.944	0.953
**TCGA-A8-A06O**	9288	11 418	1161	9808	0.906	0.947	0.926
**TCGA-A8-A06P**	10 315	11 711	934	10 894	0.957	0.947	0.952
**TCGA-A8-A06Q**	9628	10 556	272	10 365	0.936	0.929	0.933
**TCGA-A8-A06Y**	8169	9339	891	8706	0.967	0.938	0.953
**TCGA-A8-A07G**	10 177	10 770	188	11 029	0.962	0.923	0.942
**TCGA-A8-A07L**	9265	10 525	776	9840	0.950	0.942	0.946
**TCGA-A8-A082**	9271	10 496	763	10 064	0.953	0.921	0.937
**TCGA-A8-A08F**	8613	10 141	848	9318	0.927	0.924	0.926
**TCGA-A8-A091**	10 606	12 528	1246	11 116	0.940	0.954	0.947
**TCGA-A8-A09R**	10 432	12 113	1135	11 008	0.950	0.948	0.949
**TCGA-A8-A0A4**	9284	10 664	974	9824	0.958	0.945	0.952
**TCGA-A8-A0A6**	8751	9729	692	9215	0.968	0.950	0.959
**TCGA-A8-A0A9**	9532	11 604	1573	9959	0.950	0.957	0.954
**TCGA-A8-A0AD**	9286	10 333	685	9790	0.962	0.949	0.955
**TCGA-AN-A049**	9157	10 394	815	9680	0.956	0.946	0.951
**TCGA-AN-A0FW**	9786	10 559	208	10 510	0.945	0.931	0.938
**TCGA-AO-A03R**	6720	7261	287	7300	0.964	0.921	0.942
**TCGA-AO-A0JC**	9119	10 465	691	9719	0.933	0.938	0.936
**TCGA-BH-A0E2**	11 042	12 351	842	11 639	0.959	0.949	0.954
**TCGA-BH-A0E7**	9927	10 599	200	10 834	0.955	0.916	0.935

#### Precision in coding region

*I* *n-silico* validation of the variants identified for 25 TCGA RNA-Seq breast tumors was performed using the TCGA whole exome sequencing data obtained from the same breast tumors. Since whole exome sequencing primarily targets the coding region, we focused on exonic regions to compare the variants between the RNA-Seq and the whole exome-Seq from the same sample. We obtained the TCGA protected exome sequencing mutation list, which contains both germline and somatic mutations for the 25 ER+ tumor samples from the TCGA data portal (https://tcga-data.nci.nih.gov/tcga/) and compared the WES exonic variants to exonic eSNVs obtained from RNA-Seq. The number of variants detected by the eSNV-Detect using RNA-Seq data ranged from 7261 to 12 528 in the 25 ER+ samples. Of the eSNVs identified, we were able to validate 6720 to 11 042 of the variants by comparing them to the exome sequencing protected mutation list. Some of the variants that could not be validated by exome sequencing data were due to low WES coverage (≤3 total reads) and the percentage of such variants ranged from 23 to 76%. When the comparison was limited to genomic positions where WES had 4X coverage, the precision rate of eSNVs from RNA-Seq ranged from 90.6 to 96.8% (Table 2). Even though the targeted capture for WES does not work perfectly due to repetitive or complex regions, given the accuracy of the eSNV-Detect to detect variants from RNA-Seq, we believe the variants identified by RNA-Seq in the 25 ER+ tumors could also be true. However, such variants that are confidently called by RNA-Seq and do not have at least 4X coverage on exome sequencing would require further validation. Some of the not validated eSNVs from RNA-Seq could also be due to a DNA–RNA discrepancy. We compared our list of SNV discrepancies across 25 TCGA ER+ samples with two databases of known RNA-editing sites (RADAR (37) and DARNED (38)). We found 60 out of 7831 in the known RNA-editing databases. Although most of the novel variants were not identified in the database, but as indicated in Figure 2 we did see A→G and T→C enriched as the top 2 groups (Supplementary Figure S2) among all which indicated that A→I RNA-editing may potentially be responsible for part of the source of discrepancy.

#### Sensitivity in coding region

To evaluate the sensitivity/recall performance of the eSNV-Detect, we validated variants in the WES protected mutation list with RNA-Seq eSNV calls from the same sample. We investigated the coding variants that were present in TCGA exome protected mutation lists at which the nucleotide base pairs are expressed in RNA-Seq for 25 ER+ tumors (range of 7300–11 639 single nucleotide variants). Among those tumors, 6720–11 042 eSNVs were detected by eSNV-Detect and resulted in a recall rate/sensitivity ranged from 91.6–95.7% (Table 2). The majority of the false negative eSNVs were called, but were assigned a lower confidence by the eSNV-Detect method (detected by one aligner) and thus were not included in the comparison.

We investigated the frequency across the false negative eSNVs across all 25 samples (Supplementary Figure S3). It is noted that only a very small fraction of them (1.9%, 166 SNVs) were repeatedly missed as false negatives in more than 10 samples. Only 18 (0.2%) of them were repeatedly missed in more than 20 samples. It is reasonable to state that the false negatives were random instead of being caused by potential aligner bias. We further investigated the list of 131 genes associated with 166 false negative eSNVs that are observed in more than 10 samples. A functional cluster analysis of 131 genes by DAVID functional annotation tool (39) revealed the functional cluster in mitochondrion, immune response and ribosomal protein. We further investigated the overlap of 131 genes with imprinted genes or epigenes. We didn't find huge overlap between the list of genes with known imprinted genes (http://www.geneimprint.com/site/genes-by-species) (3 out of 131) and the list of known human epigenes (5 out of 131) obtained by Huether *et al*. (40). Based on our analysis, we estimate that only a small portion (1–3%) of the loss of sensitivity could be due to allele-specific expression caused by imprinted or epigenetic genes.

#### Two-aligner comparison

To check out the benefit of the two-aligner strategy, we investigated variants of different confidence levels in 25 TCGA ER+ breast tumor samples. High confident variants (validated by both aligners) achieved a precision range from 96 to 98% in the 25 ER+ tumors. Low confident variants called by MapSplice or TopHat alone had a precision of 14.6–34.0% and 12.0–22.3% respectively (Supplementary Table S6). We achieved high precision and sensitivity by obtaining eSNVs identified by multi-aligners from the eSNV-Detect workflow.

#### ts/tv ratio

Of those eSNVs validated in 25 TCGA ER+ tumor samples, the transition-to-transversion ratio (ts/tv) was ∼3.0. Over the whole genome, the ts/tv ratio is around 2.6, which is consistent with the reported expected value in the exome and the whole genome (41), suggesting a reasonable overall quality of our eSNV calling set.

#### UTR precision

Given the high coverage of UTRs from TruSeq TCGA RNA-Seq data, we also computed the precision in UTR regions. For those variants with >4X WES coverage, we were able to validate on average 93.4% of UTR variants in 25 ER+ tumors. The methods for UTR precision calculations are similar to the above precision results section for coding regions.

#### Comparisons of mutations with TCGA

In the 25 TCGA ER+ tumors we investigated, we confirmed a high mutation rate of non-synonymous variants (including both germline and somatic ones) for genes which were reported to be significantly mutated with somatic or germline variants in the TCGA paper (7), such as PIK3CA (9 out of 25), MAP3K1 (25 out of 25), TP53 (23 out of 25), CDH1 (25 out of 25), ATM (25 out of 25), BRCA1 (11 out of 25), BRIP1 (16 out 25) and others (See Table 3). Our eSNV protein domain annotations indicated that all 11 samples with BRCA1 mutations and all nine samples with FOXA1 mutations had at least one affected protein domain per gene. Although detected in only in one sample, MAP2K4, PTEN, CHEK2, RB1 and RAD51C were found to have a deleterious eSNV affected protein domain, as shown in Table 3.

**Table 3.** Gene level eSNVs summary for most frequently mutated genes listed in the TCGA paper (7)

Gene	# of samples with mutations	# of samples with mutations in protein domain	# of samples with deleterious mutations (AVSIFT)	# of samples with deleterious mutations in domain
PIK3CA	10	3	2	1
MAP3K1	25	1	3	1
GATA3	1	1	1	1
TP53	21	4	3	3
CDH1	4	4	4	4
MAP2K4	1	1	1	1
MLL3	5	2	2	0
PIK3R1	3	3	1	1
AKT1	1	1	1	1
PUNX1	1	1	1	1
CBFB	1	1	1	1
TBX3	1	0	1	1
NCOR1	4	0	3	0
CTCF	1	1	1	1
FOXA1	9	9	8	8
SF3B1	1	1	1	1
CDKN1B	9	0	0	0
RB1	1	1	1	1
AFF2	1	1	1	1
NF1	1	0	0	0
PTPN22	19	0	0	0
PTPRD	1	1	0	0
ATM	23	1	5	1
BRCA1	11	11	10	4
BRCA2	15	0	2	0
BRIP1	16	0	16	0
CHEK2	1	1	1	1
NBN	13	0	0	0
PTEN	1	1	1	1
RAD51C	1	1	1	1

#### Significant mutated gene network and pathway analysis

We selected 2599 genes with deleterious (AVSIFT < 0.05) eSNVs that are located in a protein domain from the 25 ER+ tumors. IPA pathway analysis (www.ingenuity.com, QIAGEN, Redwood City,CA) of the 2599 genes showed an altered estrogen receptor network (Supplementary Figure S1). The genes corresponding to variants altered canonical pathways such as antigen presentation pathway and OX40 signaling pathway, indicating immune and inflammation response, as well as the tRNA charging pathway which was previously reported to be associated with breast cancer (42).

### Single-cell RNA-Seq data

Single-cell transcriptomes show great transcriptional fluctuations and heterogeneity because of dynamic changes during the cell cycle, which can be characterized by single-cell mRNA-Sequencing (43). The single-cell mRNA-Seq and the traditional GA-II mRNA-Seq data from MDA-MB-231 cancer cell line were obtained and the eSNVs were called using the eSNV-Detect pipeline as described in ‘Materials and Methods’ section (Supplementary Table S7).

**Figure 4.**
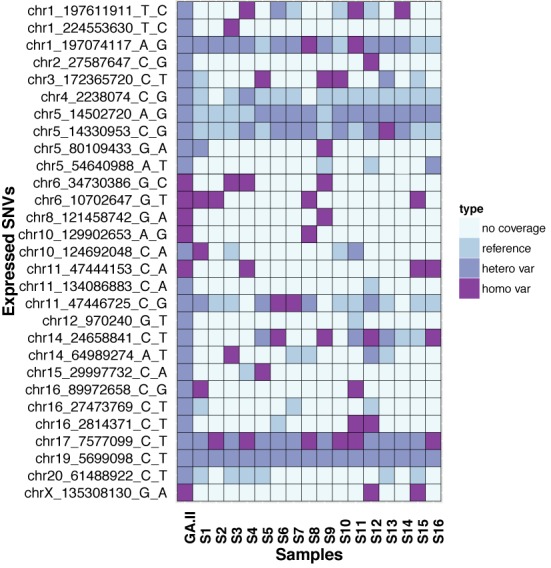
Apply eSNV-Detect to Single Cell Sequencing and the matching multicellular GA-II RNA-seq. The comparison between the single-cell data and the multicellular data shows the celluar heterogeneity of the single cells in variant calling.

To show the ability of the eSNV-Detect pipeline to capture the diversity of variant calls among single-cells, we obtained a list of 102 unique mutations that had been reported in the COSMIC database for MDA-MB-231 cell lines (28). Of the 102 mutations, only 29 mutations were observed in at least one of the single-cell samples and called confidently in the Illumina GAII traditional transcriptomic data. The zygosities of the eSNVs calls were investigated by the eSNV-Detect pipeline and the findings are shown in Figure 4. It can be seen that variants with homozygous alternative alleles in traditional GA-II sequencing data were found also to be homozygous, when coverage was sufficient. In contrast variants with heterozygous alleles in traditional GA-II sequencing data could be found to be homozygous reference, heterozygous variants or homozygous variants in single cell preparations. Even with the low sequencing depth of MiSeq, the eSNVs from single-cell mRNA-Seq data showed the heterogeneity of eSNV calls successfully in the single-cells. The same heterogeneity pattern was also observed in a Hi-Seq data set of 89 MDA-MB-231 single cells (the analysis of that data is currently ongoing for gene expression, fusion transcripts, eSNVs and hence not included as part of this manuscript).

## DISCUSSION

At present, there are software analysis methods such as GATK (44), VARSCAN (45) samtools mpileup (46) that are publically available for DNA variant analysis from whole genome or whole exome sequencing datasets. Most genotype calling algorithms are designed for calling mutations from DNA data rather than mRNA data. Current sequencing workflow such as GATK best practices (44) also focus on DNA variant calling and do not have a direct approach for extracting expressed variants from RNA-Seq. Identification of SNVs from RNA-Seq is still challenging because of the dynamic range of gene expression, splicing and translocations. To our knowledge, there are only few methods (SNVMix (17), SNPiR (18)) to call variants from RNA-Seq data. Even these methods that currently exist for calling eSNVs are either specific for calling variants from cancer (17) or calling variants without annotation or prioritization.

We have developed the eSNV-Detect, a comprehensive bioinformatics pipeline to call variants with high precision and recall rates, which annotates and prioritizes based on genomic and proteomic domains. The eSNV-Detect pipeline uses a combination of open access bioinformatics tools, with several customizations and in-house developed methods, to identify eSNVs from RNA-Seq data. Sequence characteristics such as number of reads at a nucleotide position, reference supporting reads, alternate allele reads, location of variant in the reads, forward strand supporting reads, reverse strand supporting reads, base qualities, mapping qualities etc. are used to call eSNVs. Systemic mapping errors such as strand bias in sequencing, PCR duplicates and/or multi-mapping errors do exist in RNA-Seq technologies. Efforts to account for these minimize the false discovery rate of variants thereby controlling the Type I error rate of eSNV calls. In addition to sequence characteristics individual aligners have their own strengths and weaknesses in terms of aligning junction reads from RNA-Seq data. Hence, our strategy to use at least two complementary aligners to assign confidence score facilitates prioritization of candidate eSNVs for further clinical interrogation. The eSNV calls with evidence found in both aligners and callers increases confidence. Further, when sensitivity is more of a concern, taking the union of the variants identified from both aligners would ultimately help the selection of the eSNVs missed because of aligner bias. However, the two-aligner concept does require additional resources to call eSNVs but it is predicated due to the stringent criteria required for clinical settings. The eSNV-Detect pipeline is freely available and can be downloaded on a windows machine with virtual machine concept or as a stand-alone UNIX machine or a parallel sun grid machine.

The calls from the eSNV-Detect method were validated using three independent RNA-Seq datasets with three different analysis using SNP chip data, exome sequencing data and Sanger sequencing. The method was first tested using a lymphoblastoid sample for which we have RNA-Seq and Human Omni2.5 SNP chip data. We have then applied our method to 25 ER+ breast cancer samples from TCGA project for which we have both RNA-Seq and exome sequencing data. As described in the ‘Results’ section, precision and recall rates >90% were achieved for both analyses. Furthermore, we have validated 29/31 eSNV candidates for an ER+ breast tumor for which we have tumor and adjacent normal tissue using the Sanger sequencing. In addition, the eSNV-Detect pipeline was applied to 16 single-cell breast cancer cell line RNA-Seq data (MiSeq) and observed heterogeneous genotype calls, even at low read depths, for a set of somatic mutations obtained from COSMIC database (28).

Calling of variants from RNA-Seq has a number of applications, such as it allows for the validation of germline or somatic variants called by whole exome or whole genome sequencing. Further, RNA-Seq enables the detection of previously unidentified variants that are functionally important such as UTRs and non-coding RNAs, which are difficult to capture using targeted exome sequencing. For example, our study of 25 TCGA ER+ tumor eSNV calls from RNA-Seq data demonstrate an increase in the number of variants called by an average of 6% in contrast to exome sequencing data. This confirms that there are additional variants obtained from RNA-Seq data, compared to exome sequencing data.

At present, large-scale projects like TCGA consist of both exome and RNA-Seq data available for same individuals. Thus far, the genotyping in the TCGA datasets were performed using exome sequencing and SNP arrays and hence do not take complete benefit of existing RNA-Seq data. Our ability to understand the complexity of genotype-phenotype relationship in these tumors relies on the effective identification of genomic variants in tumor samples. Hence, we are currently processing TCGA RNA-Seq data using the eSNV-Detect pipeline, and this effort is currently ongoing.

The eSNV-Detect has a high precision rate and we have thus far applied our method to large scale tumor RNA-Seq samples, time series datasets and individualized medicine projects at the Mayo Clinic. For individualized medicine projects, where we have both RNA-Seq and Exome-Seq data, we successfully validated candidate eSNVs with a high accuracy rate. In the genomic medicine setting where the clinical treatment decisions are crucial eSNV-Detect has been successful in identifying variants confidently.

## SUPPLEMENTARY DATA

Supplementary Data are available at NAR Online.

SUPPLEMENTARY DATA
