# Supplementary material for: The eSNV-detect: a computational system to identify expressed single nucleotide variants from transcriptome sequencing data
Source: Nucleic Acids Res. 2014 Oct 28;42(22):e172. doi: 10.1093/nar/gku1005 (PMC4267611; doi:10.1093/nar/gku1005)
Supplement: SUPPLEMENTARY DATA [file supp_42_22_e172__index.html]

The eSNV-detect: a computational system to identify expressed single nucleotide variants from transcriptome sequencing data — The eSNV-detect: a computational system to identify expressed single nucleotide variants from transcriptome sequencing data — The eSNV-detect: a computational system to identify expressed single nucleotide variants from transcriptome sequencing data — SUPPLEMENTARY DATA 

# The eSNV-detect: a computational system to identify expressed single nucleotide variants from transcriptome sequencing data

## SUPPLEMENTARY DATA

**Files in this Data Supplement:**

- SUPPLEMENTARY DATA
- SUPPLEMENTARY DATA
- SUPPLEMENTARY DATA
- SUPPLEMENTARY DATA
- SUPPLEMENTARY DATA
- SUPPLEMENTARY DATA
- SUPPLEMENTARY DATA
- SUPPLEMENTARY DATA
- SUPPLEMENTARY DATA
