# Supplementary material for: The eSNV-detect: a computational system to identify expressed single nucleotide variants from transcriptome sequencing data
Source: Nucleic Acids Res. 2014 Oct 28;42(22):e172. doi: 10.1093/nar/gku1005 (PMC4267611; doi:10.1093/nar/gku1005)

Supplementary Figure S1. The pathway network by IPA pathway analysis on 846 significantly mutated genes showed an altered estrogen receptor network. Red indicates gene in the list, no filling colors indicates gene not in the list. Rectangular shape indicates receptor, ellipse indicates transcription regulator, triangle indicates phosphatase, rhombus indicates enzyme, trapezoid indicates transporter and double circle indicates complex or group.

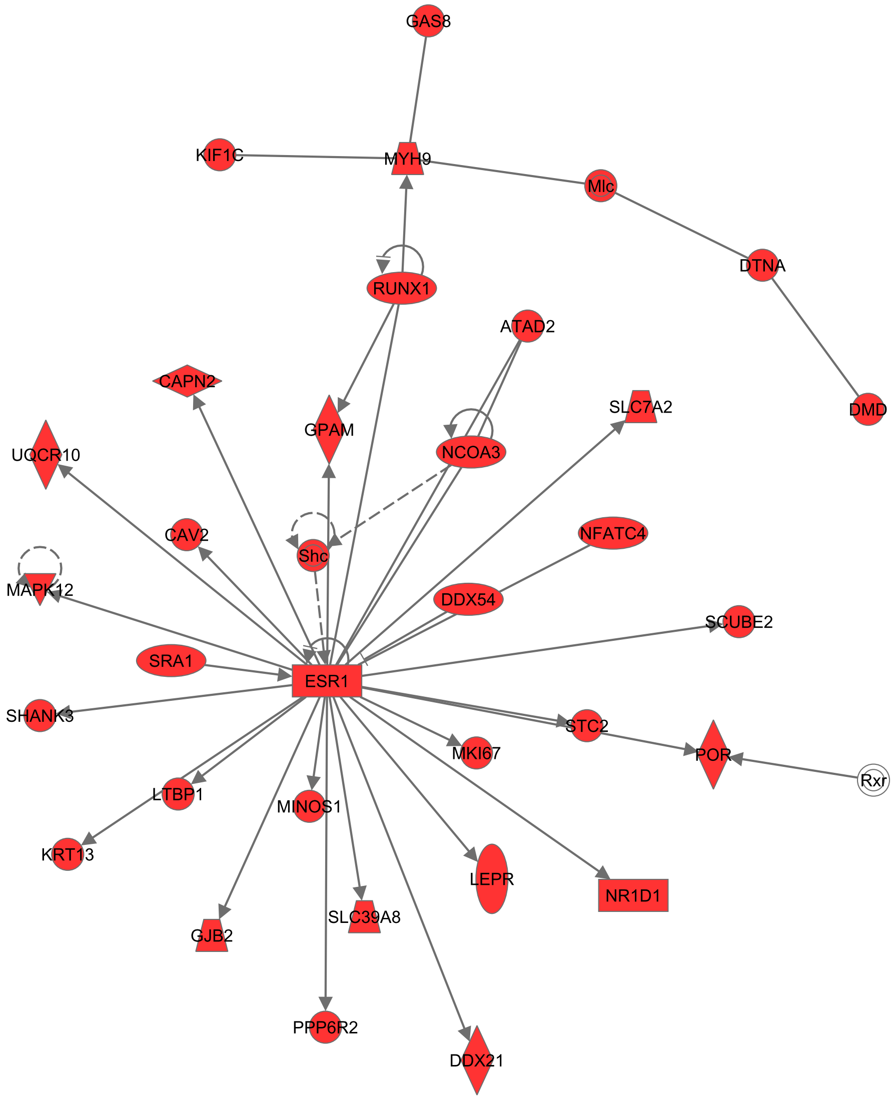

© 2000-2014 Ingenuity Systems, Inc. All rights reserved.

Supplementary Figure S2. Histogram of the distribution of the non-called variants across 25 TCGA ER+ samples

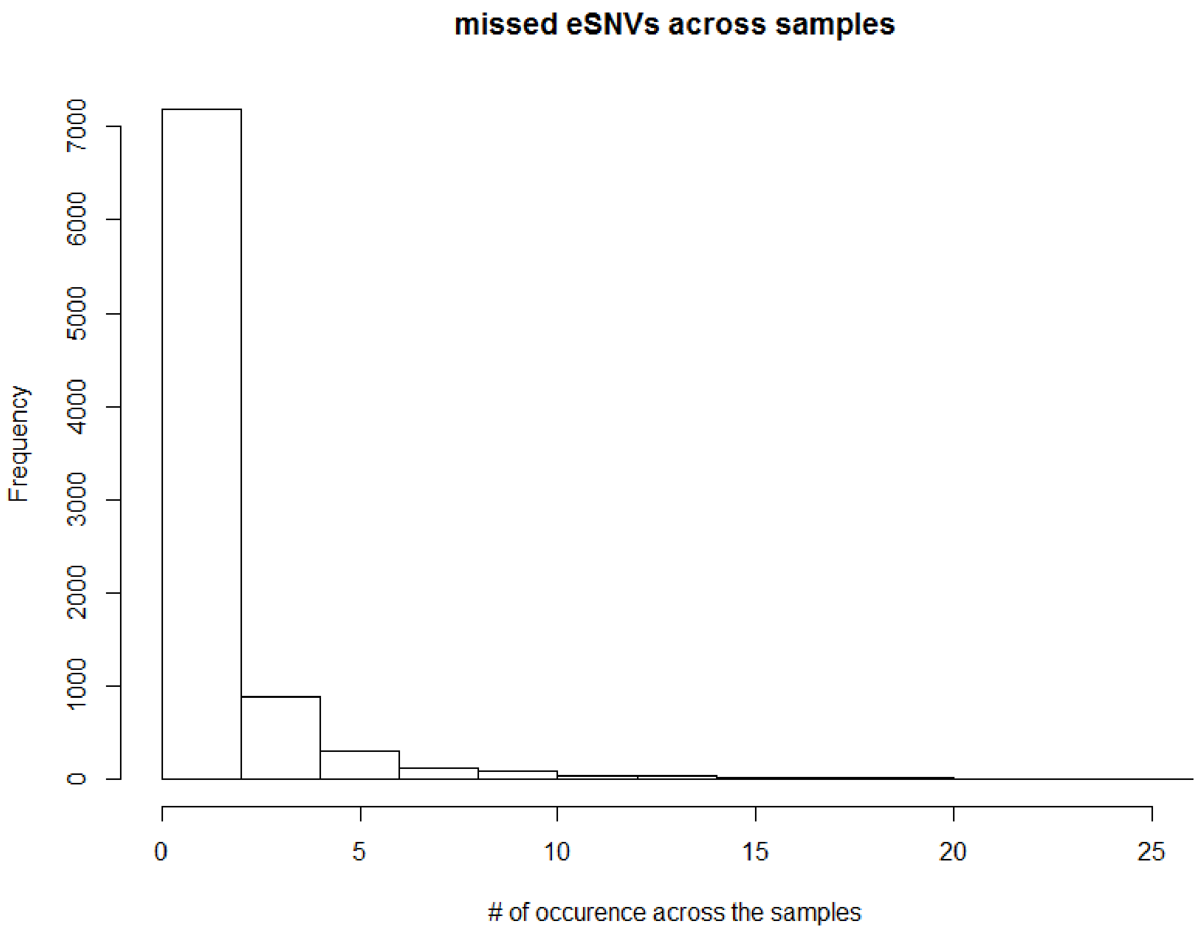

Supplementary Figure S3: Nucleotide changes of DNA-RNA discrepancy SNVs

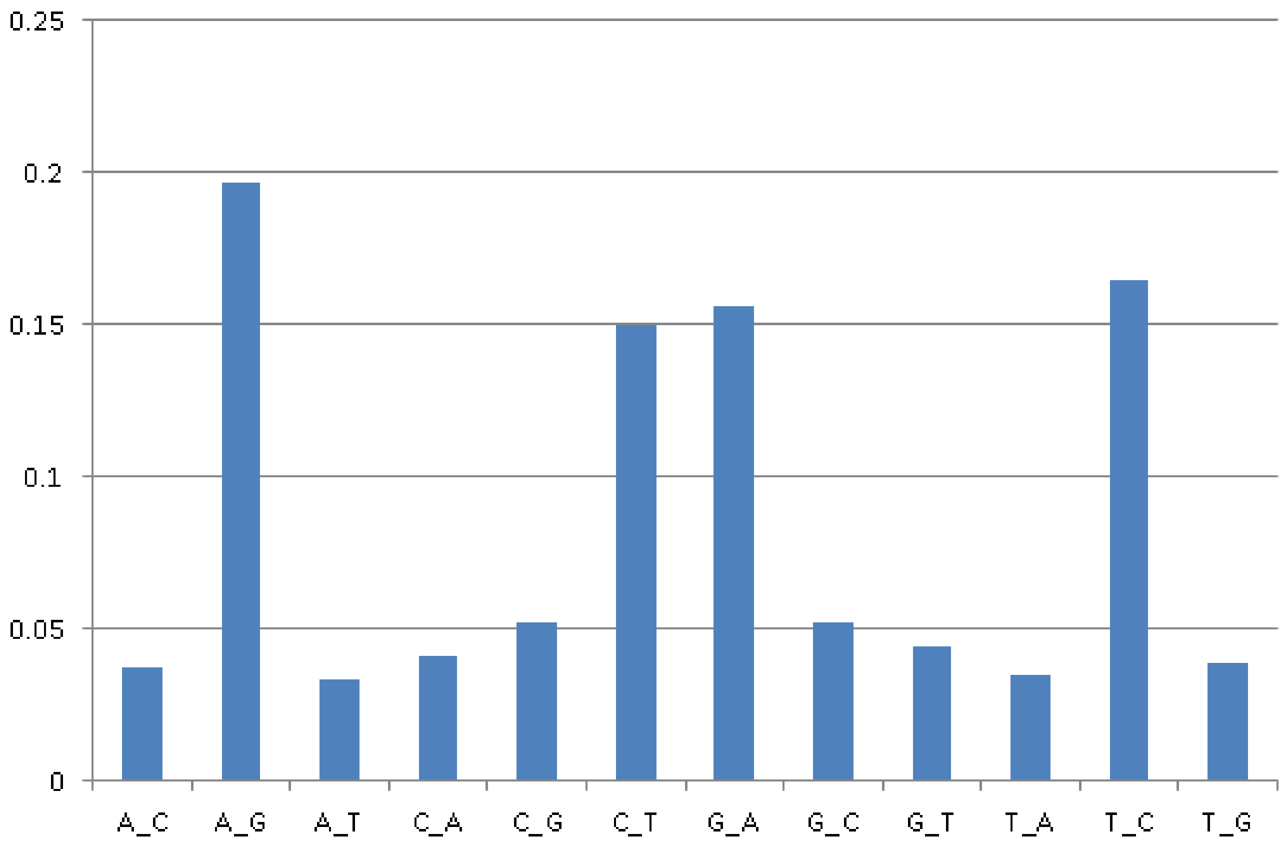

Supplement: SUPPLEMENTARY DATA [file supp_gku1005_nar-01266-met-n-2014-File007.pdf]
