# Supplementary material for: The eSNV-detect: a computational system to identify expressed single nucleotide variants from transcriptome sequencing data
Source: Nucleic Acids Res. 2014 Oct 28;42(22):e172. doi: 10.1093/nar/gku1005 (PMC4267611; doi:10.1093/nar/gku1005)
Supplement: SUPPLEMENTARY DATA [file supp_gku1005_nar-01266-met-n-2014-File006.docx]

**Supplementary Methods**

1. For all analyses described in the manuscript, the sequencing data sets were aligned against the human genome (release NCBI GRCh37.1b).

2. The RNA-Seq aligner combinations used for three different analyses that were described in the manuscript are explicitly listed below. For each of the analysis, all running parameters used were default as explained in the next section unless otherwise stated.

**For Lymphoblastoid cell line (NA07347)** **analysis** we used Tophat2 and BWA as our option of aligners for the eSNV-Detect analysis.

**For 25 TCGA ER+ breast tumor eSNV-Detect analysis,** we used the TCGA bam files that have been distributed through the CGHUB <https://cghub.ucsc.edu/software/downloads.html> via Gene Torrent software. These bam files were previously aligned with MapSplice software. For the second aligner, we converted the bam files obtained from TCGA to fastq files and then re-aligned using TopHat1.3 version (that was available at the time of analysis).

**The eSNV-Detect analysis of Single cells** was performed using Tophat2 and BWA aligners for MiSeq data. Because the read depth of MiSeq was low, we reduced the threshold of required alternative allele read depth to 2.

3. The default running parameters for the eSNV-Detector method are described below (also listed in Table 1):

During samtools mpileup and bcftools raw variant calling, the filtering criteria of base quality >13 and mapping quality >20 were used

Nucleotide positions with less than 4X coverage or 4 alternative allele supporting reads were eliminated from variant analysis.

For variants with total coverage less than 100, we required the ratio of reads Ratio_i_ to be greater than 0.1; while for variants with higher coverage (more than or equal to 100), the Ratio_i_ threshold should be 0.05.

For variants with total coverage less than 100, the strand bias ratio (SBS_i_) of > 0.1 was required and for variants with greater than 100x coverage a SBS_i_ of > 0.05 was preferable.

ReadRankPosSum (RRPS) score was obtained using the GATK. A recommended RRPS score threshold of (-8.0, 8.0) from GATK was used in our method.
